# Regulation of metabolic networks by small molecule metabolites

**DOI:** 10.1186/1471-2105-8-88

**Published:** 2007-03-13

**Authors:** Alex Gutteridge, Minoru Kanehisa, Susumu Goto

**Affiliations:** 1 Bioinformatics Center, Institute for Chemical Research, Kyoto University, Kyoto, Japan 611-0011

## Abstract

**Background:**

The ability to regulate metabolism is a fundamental process in living systems. We present an analysis of one of the mechanisms by which metabolic regulation occurs: enzyme inhibition and activation by small molecules. We look at the network properties of this regulatory system and the relationship between the chemical properties of regulatory molecules.

**Results:**

We find that many features of the regulatory network, such as the degree and clustering coefficient, closely match those of the underlying metabolic network. While these global features are conserved across several organisms, we do find local differences between regulation in *E. coli *and *H. sapiens *which reflect their different lifestyles. Chemical structure appears to play an important role in determining a compounds suitability for use in regulation. Chemical structure also often determines how groups of similar compounds can regulate sets of enzymes. These groups of compounds and the enzymes they regulate form modules that mirror the modules and pathways of the underlying metabolic network. We also show how knowledge of chemical structure and regulation could be used to predict regulatory interactions for drugs.

**Conclusion:**

The metabolic regulatory network shares many of the global properties of the metabolic network, but often varies at the level of individual compounds. Chemical structure is a key determinant in deciding how a compound is used in regulation and for defining modules within the regulatory system.

## Background

Cellular metabolism comprises all the chemical reactions that take place within a cell. Through these various reactions, the cell generates biomass and energy, replicates itself, and can transmit information to its neighbours. Metabolic pathways and networks are formed from linking individual reactions into ever more complex, higher order structures. In recent years, our increasingly complete knowledge of the individual component reactions has revealed some of the emergent properties of these higher order networks[1-3].

A fundamental property of all organisms is their ability to adapt to changing environments. From a yeast cell in a fermentation reactor, to a human engaging in exercise, an organism must be able to regulate its metabolism in order to adapt to changes in its environment. Cells use a number of mechanisms to regulate their metabolism. Two of the most common and well studied are genetic regulation (repression or activation of enzyme gene transcription)[4], and enzyme inhibition/activation by small molecules (allosteric inhibition for example)[5], though other methods of regulation, such as mRNA attenuation[6,7], riboswitches[8] and cellular compartmentalisation[9], also have important roles to play.

In many systems several of these regulatory processes are used together to provide a range of metabolic responses[10]. The *Escherichia Coli trp *regulon, for example, demonstrates control by genetic regulation, mRNA attenuation and enzyme inhibition. In a classic feedback loop, tryptophan inhibits the enzymes, and the production of those enzymes, that are required for its synthesis[11].

Previously, Barrett *et al*[12] studied the genetic regulation of metabolism in *E. coli *using a genome-scale model of the known metabolic and genetic regulatory networks[13]. Similarly, Yeang and Vingron[4] examined the way in which metabolites exert feedback control over enzyme gene expression. In contrast, although the role of enzyme inhibition and activation in individual pathways has been studied and modelled in some detail, genome-scale analyses of enzyme inhibition and activation networks have been lacking to date. In this paper we present such an analysis.

The advantage of genome-scale studies is that they may identify emergent properties of the system that are not obvious at the reaction, or even pathway, level. One such emergent property of many biological (and non-biological) networks, including metabolic networks, is their scale free topology[14]. It has been proposed that this property is biologically useful and actively selected for by evolution[15], though other studies have questioned whether this is a real feature of metabolic networks[16], and have suggested that the topology simply derives from the way in which new enzyme functions evolve. In this study of metabolic regulatory networks, we expect to see networks that closely follow the topology of the underlying metabolic network and to see the global properties of the networks conserved across different organisms, though there maybe local variations between organisms.

Enzyme inhibition/activation is also the basis for the efficacy of many drugs. In particular, modern *in silico *drug design has often focussed on designing compounds capable of inhibiting disease associated enzymes. HIV protease inhibitors are some of the best known examples of this type of drug[17]. However, being able to make *in silico *predictions about which enzymes a compound will bind to has proved to be a difficult problem[18,19]. It is an important question, because knowing whether a compound interacts with one, or a whole set of enzymes is important for predicting potential side-effects. By looking at how groups of chemically similar compounds regulate similar enzymes we are able to see how Nature uses this sort of regulatory 'cross-talk' to form functional modules within the larger network, and even make simple predictions of novel regulatory interactions. We can also examine which compounds are commonly used as regulatory molecules and whether these compounds have chemical properties which distinguish them from molecules used less often.

In summary, this study aims to answer basic questions about how the enzyme inhibition and activation regulatory system is organised. This includes examining the topology of the overall regulatory network in several organisms, investigating which compounds are used as regulators and the relationship between chemical structure and enzyme inhibition. A full understanding of metabolism and its regulation will require merging models of genetic regulation with enzyme inhibition/activation and other regulatory mechanisms. This full model is still someway distant, not least because the data describing these processes is often incomplete. However, we hope that this study goes someway towards the goal of a full and complete understanding of cellular metabolism.

## Results

Inhibitory and activatory interactions between small molecules and enzymes were extracted from the BRENDA database[20] for *Escherichia coli*, *Homo sapiens*, *Plasmodium falciparum *and *Saccharomyces cerevisiae *as described in the methods section. The total number of extracted interactions and other details were calculated [see Additional file 1]. In brief, the *E. coli *network comprises 1847 compound/enzyme interactions, and the *S. cerevisiae *and *H. sapiens *networks comprise 1462 and 1435 interactions respectively. The dataset for *P. falciparum *is smaller, comprising 599 interactions.

Inhibitor/activator data was also downloaded from EcoCyc[21] to provide a comparison dataset. After processing, the EcoCyc data representing regulation in *E. coli *comprises 667 compound/enzyme interactions.

### Network Properties

The regulatory interactions for each organism are initially represented by a directed bipartite network. In a bipartite network the set of nodes can be divided into two disjoint sets, and each edge connects a node from one set with a node from the other set. In our network the nodes are either compounds or enzymes. An edge is drawn to connect a compound node to an enzyme node when the compound is known to regulate the enzyme. Edges are labelled according to whether the regulation is inhibitory or activatory.

First we plot the network's degree distribution. The degree of a node (*k*) is a measure of how many edges are connected to that node (in this network, for the compound nodes this corresponds to how many EC codes a compound regulates). We count the degree of each compound node and bin the data into bins of exponentially increasing size. The probability of a compound being in each bin (*P*(*k*)) is then calculated for each bin. *P*(*k*) is the number of nodes in a given bin divided by the total number of nodes.

**Figure 1:**
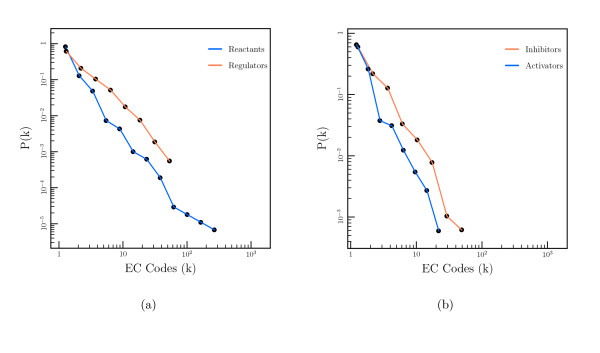
(a) Log-log plot of the degree distribution for compound nodes in the regulatory and metabolic networks of *E. coli*. Colors: (orange) Regulatory network, (blue) metabolic network. (b) Log-log plot of the degree distribution for compound nodes in the regulatory network divided into activating compounds and inhibiting compounds. Colors: (orange) Inhibitors, (blue) activators.

A log-log plot of *P*(*k*) against *k *for the interactions taken from *E. coli *is shown in Figure 1(a). For comparison, the degree distribution for compounds in the underlying metabolic network is also shown. In this network a compound node is connected to an enzyme node if the compound is a substrate or product (rather than regulator) of the enzyme. The metabolic network information is extracted from KEGG[22] as detailed in the methods section.

The data is fitted to a power law distribution, as shown in Equation 1, using a maximum likelihood estimation method.

*P*(*k*) = *k*^-*γ *^    (1)

The fitted exponent for the underlying metabolic network is 2.19, very close to the value reported by Jeong *et al *who used an essentially identical method[3]. The fitted exponent for the regulatory data is 1.64 with 95% confidence limits for the fit of 1.58–1.70. This value is significantly smaller than that found for the metabolic network (and many other biological networks), where the value is often 2–3. The same analysis on the EcoCyc regulatory data gives a fitted exponent of 1.79 ± 0.10. This is larger than the value found from BRENDA, but still appears significantly smaller than the exponent of the metabolic network.

The regulatory compounds in BRENDA are split into those compounds that activate an enzyme and those that inhibit it. The number of inhibitory interactions (1497) is greater than the number of activatory interactions (350), but we can still compare the networks formed by each. The degree distribution for inhibitors and activators from *E. coli *is shown in Figure 1(b). The degree exponent for the inhibitors is 1.68 ± 0.07, whilst for the activators it is 1.94 ± 0.13, indicating a small, but significant, difference in the degree distribution between inhibitors and activators.

There is also evidence for a high degree of modularisation in metabolic networks. This is most obviously represented by the abstraction made by scientists when they divide portions of the network into separate pathways as found in KEGG and EcoCyc[22,21]. In addition, there is evidence for considering the metabolic network as fundamentally modular[23]. Whilst a modular and scale-free topology seems contradictory (since the presence of modules implies a fundamental scale) a hierarchical network model has been proposed that can have both properties[24].

The characteristic feature of a hierarchical network is that the clustering coefficient is smaller for highly connected nodes than for loosely connected nodes. The clustering coefficient for a node is defined as the proportion of links between its adjacent nodes divided by the number of links that could possibly exist. In practical terms the spoke nodes tend to form tightly connected modules (corresponding to pathways in the metabolic network or regulatory modules in the regulation network) while the hub nodes connect these modules to each other. A log-log plot of *C*(*k*) (defined as the average clustering coefficient for nodes of degree *k*) against *k *should be linear with a slope approaching -1 for a hierarchical network.

**Figure 2:**
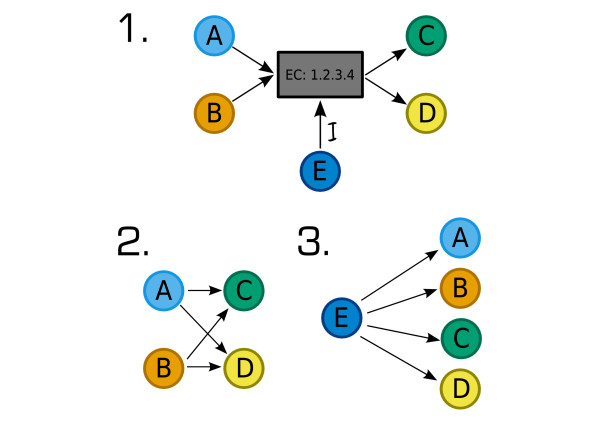
An example of a reaction represented first by a bipartite network and second by two monopartite networks. The compounds A and B are substrates of reaction catalysed by EC: 1.2.3.4, C and D are products and E is an inhibitor of EC: 1.2.3.4. In network 2. only the metabolic network is used and substrates are connected to products. In network 3. only the regulatory network is used and the regulator (E) is connected to each of the substrates and products, representing the fact that E regulates the levels of these compounds in some way. Note that E could be one of the products or substrates of the reaction, in which case it is connected to itself.

The original bipartite networks (comprising separate enzyme and compound nodes) used above are transformed into monopartite networks comprising just compounds. In the metabolic network, compounds that are substrates of a given enzyme are connected to the product compounds of that enzyme. In the regulatory network, compounds that are regulators of a given enzyme are connected to the product and substrate compounds of that enzyme. An example of a reaction and its transformation into the monopartite networks used below is shown in Figure 2.

**Figure 3:**
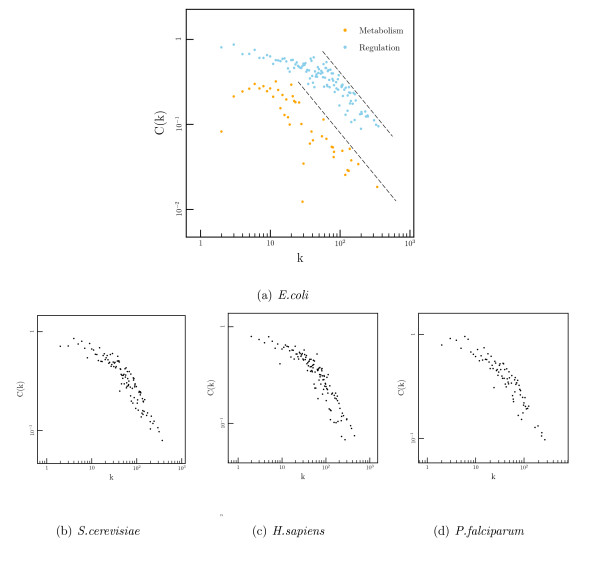
(a) Log-log plot of *C*(*k*) against *k *from metabolic and regulatory networks in *E. coli*. *k *is the outgoing degree of each node. *C*(*k*) is defined as the average clustering coefficient (*C*) of all the nodes of a given *k*. The dashed lines correspond to *C*(*k*) ~ *k*^-1^. Colors: (orange) metabolic network, (blue) regulatory network. (b-d): *C*(*k*) against *k *from the regulatory networks in *S. cerevisiae*, *H. sapiens *and *P. falciparum*.

Figure 3 shows the log-log plots of *C*(*k*) against *k *for both of these networks. The metabolic network shows the same behaviour as observed previously[24], with higher degree nodes showing the predicted linear relationship between *k *and *C*(*k*). This relationship breaks down at *k *< 10 below which the clustering coefficient levels off. The regulatory network shows similar behaviour, but with several differences: Firstly, the average clustering coefficient in the regulatory network is around double the coefficient in the underlying metabolic network. Secondly, though again there is a portion of the network that appears to follow the *C*(*k*) ~ *k*^-1 ^law this regime only begins at *k *≈ 60. Below this value the slope becomes less steep.

These results would suggest that the regulatory network is more clustered than the metabolic network (due to the high clustering coefficient) and also that the characteristic size of the modules may differ (due to the difference in the point at which the linear *C*(*k*) ~ *k*^-1 ^relationship breaks down).

### Comparing Regulation Between Organisms

**Figure 4:**
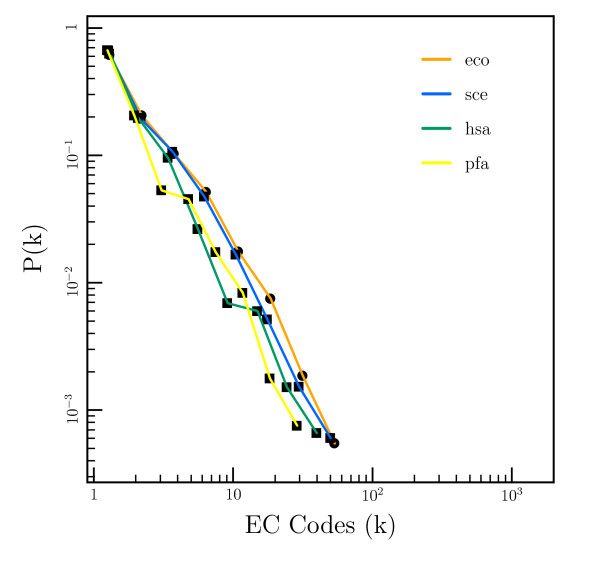
Log-log plot of the degree distribution for compound nodes in the regulatory network from several different organisms. Colors: (orange) *E. coli*, (blue) *S. cerevisiae*, (green) *H. sapiens*, (yellow) *P. falciparum*.

To compare the network between different organisms, we plot the same log-log plot as in Figure 1(a), using the data from each of the organisms studied. This is shown in Figure 4. The fitted degree exponent for each organism is given in Table 1. Note that no allowance is made for the multi-cellular nature of *H. sapiens*. Different tissues and cells within *H. sapiens *will often be controlled under different regulatory systems since different isoforms of the same enzyme may be expressed.

**Table 1:** Degree exponents for the compound nodes in the regulatory networks of *E. coli*, *S. cerevisiae*, *H. sapiens *and *P. falciparum*.

Organism	Degree Exponent
*E. coli*	-1.64 ± 0.06
*S. cerevisiae*	-1.68 ± 0.07
*H. sapiens*	-1.81 ± 0.07
*P. falciparum*	-1.82 ± 0.11

Although there is some variation between the organisms, the overall distribution of regulatory interactions remains similar. The slightly higher degree exponent for *H. sapiens *implies that highly connected hub compounds form a lower proportion of the total in *H. sapiens *than in *E. coli *or *S. cerevisiae*. It also appears that *P. falciparum *has evolved to have a regulatory network more similar to its host, *H. sapiens *than the other single celled organisms. To investigate these differences further, we looked for more local changes in the compounds involved in regulation in each organism. For *E. coli*, *S. cerevisiae*, *H. sapiens *and *P. falciparum *the top ten most commonly observed regulatory compounds are shown in Table 2.

**Table 2:** The ten compounds observed most commonly acting as regulators in *E. coli*, *S. cerevisiae*, *H. sapiens *and *P. falciparum*.

	*E. coli*		*S. cerevisiae*		*H. sapiens*		*P. falciparum*	
Rank	Compound	Interactions	Compound	Interactions	Compound	Interactions	Compound	Interactions
1	ATP	70	ATP	65	ATP	51	ATP	56
2	ADP	70	ADP	52	Dithiothreitol	51	ADP	54
3	AMP	55	Orthophosphate	51	ADP	40	AMP	41
4	Orthophosphate	53	AMP	50	Orthophosphate	39	Orthophosphate	30
5	Mercaptoethanol	40	GTP	31	Mercaptoethanol	39	Pyrophosphate	29
6	Pyrophosphate	38	Pyrophosphate	31	AMP	31	GTP	28
7	GTP	35	Fe^2^+	26	Glutathione	30	CTP	23
8	Fe^2^+	35	NADH	25	Pyrophosphate	24	UTP	23
9	Glutathione	29	UTP	25	UTP	23	Adenosine	20
10	NADH	28	Glutathione	24	GTP	22	GDP	20

We can clearly see the importance of ATP and related metabolites in metabolic regulation from this data. A similar trend was seen in the data from EcoCyc, with ATP, ADP and AMP forming the top three regulator compounds [see Additional file 1]. This reflects the importance of ATP and related compounds in the underlying metabolic network. However, some of the other commonly seen regulators, such as glutathione and mercaptoethanol, are less important metabolically. These compounds often contain reducing elements such as thiol groups which can activate or inhibit many enzymes in a non-specific manner by reducing or oxidising active site cysteine residues.

Although the same compounds are consistently observed as the most important across all the organisms studied, there are some compounds whose importance varies considerably. To compare the relative importance of a compound in different organisms, we rank each compound by the number of ECs it regulates in each organism. In Table 3 we show those compounds undergoing the largest change in rank between *E. coli *and *H. sapiens*. Note that we only consider compounds present as metabolites in both organisms, so there are some small changes to the ranks shown in Table 2.

**Table 3:** Comparison of those compounds that have different importance in regulation between *E. coli *and *H. sapiens*.

		Rank		Interactions	
No.	Compound	*E. coli*	*H. sapiens*	*E. coli*	*H. sapiens*
	*More common in E. coli*				

1	Pyruvate	10	26	25	4
2	Phosphoenolpyruvate	15	28	19	2
3	2-oxoglutarate	16	27	18	3
4	Pyridoxal phosphate	15	26	19	4
5	D-fructose 1,6-bisphosphate	14	25	20	5
6	ITP	18	28	15	2
7	Succinate	15	25	19	5
8	Oxaloacetate	20	28	13	2
9	L-alanine	21	28	12	2
10	L-serine	20	27	13	3

	*More common in H. sapiens*				

10	Nitric oxide	30	24	3	6
9	3'-5'-cyclic AMP	29	23	4	7
8	Phosphatidylethanolamine	28	22	5	8
7	UTP	12	7	22	23
6	Phosphatidate	30	23	3	7
5	Ethanol	25	18	8	12
4	1-acyl-glycero-3-phosphocholine	32	22	1	8
3	CDP	28	17	5	13
2	Phosphatidylserine	30	17	3	13
1	Phosphatidylcholine	28	13	5	17

The number of interactions (EC numbers regulated) for each compound in the two organisms is shown in the right most columns. The compounds are then ranked in each organism by the number of interactions. The ranks for each compound in the two organism are shown in the middle columns. The ten compounds showing the largest decrease in rank (*i.e*. those that are more important in *H. sapiens *and the ten compounds showing the largest increase in rank (more important in *E. coli *are shown.

The compounds found more commonly acting as regulators in *E. coli *are components of glycolysis, amino acid metabolism and in particular the citric acid cycle. In contrast, the compounds found more commonly acting as regulators in *H. sapiens *tend to be phospholipids and other signalling molecules such as nitric oxide and cyclic AMP. Phosphatidylethanolamine, phosphatidate, phosphocholine, phosphatidylcholine and phosphatidylserine are all involved in glycerol/glycerophospholipid metabolism and the phosphatidylinositol signaling system.

The compound with the largest change between *E. coli *and *H. sapiens *is pyruvate which only regulates four different enzymes in *H. sapiens *compared to 25 in *E. coli*. This results in a drop in importance of pyruvate from the 10th most important regulator of metabolism in *E. coli *to the 26th most important in *H. sapiens*. We also observe that only one of the four *H. sapiens *enzymes regulated by pyruvate is also regulated by pyruvate in *E. coli*.

We performed the same analysis comparing *S. cerevisiae *and *P. falciparum *with *H. sapiens*. However, similar patterns of differences were found in each case: more regulation by glycolysis and citric acid cycle compounds in the unicellular species and more regulation by phospholipids and cell signalling compounds in *H. sapiens*.

### Types of Compounds Used As Metabolic Regulators

Since we have shown that the regulatory network has a similar overall structure and topology to the underlying metabolic network, and that common regulatory compounds like ATP are also often the most common metabolites, it is interesting to ask whether the regulatory network simply shadows the metabolic network. Is the frequency with which we see a compound acting as a regulator straight-forwardly related to the frequency with which we see it in metabolism?

**Figure 5:**
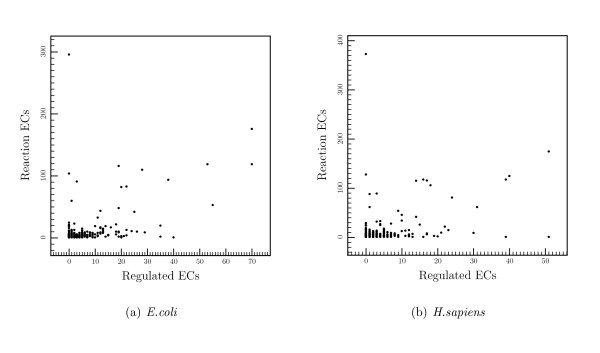
Correlation of metabolic importance with regulatory importance. In each graph each point represents a compound with the number of ECs for which it is a reactant shown on the y-axis and the number of ECs for which it is a regulator shown on the x-axis. The correlation coefficients are 0.53 and 0.42 for *E. coli *and *H. sapiens *respectively (P-values: 1.5^-58 ^and 3.9^-84^).

The relationship between the number of reactions each compound is involved in and the number of reactions regulated by each compound is shown for *E. coli *and *H. sapiens *in Figure 5. The Pearson correlation coefficient between the two values shows that the correlation is weak: 0.53 for *E. coli *and *S. cerevisiae *(not shown) and 0.42 for *H. sapiens *(Spearman's rho values are 0.44 and 0.46 for *E. coli *and *H. sapiens*; P-values are 1.5 × 10^-58 ^and 3.9 × 10^-84 ^respectively). Removing ATP/ADP from the data set emphasises the weakness of the correlation. doing so immediately lowers the Pearson coefficients to 0.40 and 0.31 in *E. coli *and *H. sapiens *respectively. This implies that, although the metabolic importance of a compound does have an effect on its use as a regulator, there are other important considerations. The fact that the relationship is slightly stronger in *E. coli *and *S. cerevisiae *may be significant. We have already seen above that *E. coli *is more heavily regulated by core metabolites than *H. sapiens*. Since the core metabolites are involved in more reactions than other compounds, the weaker correlation between metabolic importance and regulatory importance observed in *H. sapiens *also reflects this observation.

An example of two compounds with differing regulatory proclivities is ATP and NAD^+^. Both compounds are involved in many different reactions. In *E. coli*, ATP is involved in 179 different EC reactions compared to 116 for NAD^+ ^(a 54% difference), yet the number of reactions regulated by ATP is 70 compared to 19 by NAD^+ ^(a 268% difference). Possible reasons for this difference are proposed in the discussion.

Some other trends in the use of different compounds are obvious: many small molecules, for instance, are rarely used as regulators even though they are common metabolites. Nitric oxide is an example of a small compound that is used as a regulator. It regulates 6 enzymes in *H. sapiens *and 3 in *E. coli *though it is only involved metabolically in 1 reaction in either organism. In contrast, almost all the other small molecules that are highly involved in metabolism rarely act as regulators: Oxygen (2 ECs regulated/23 EC reactions), carbon dioxide (1 EC regulated/60 EC reactions) and ammonia (3 ECs regulated/91 EC reactions). Having said this, no correlation could be found between regulatory proclivity and mass for the regulator compounds in our dataset.

To find other compounds with a particularly low or high regulatory proclivity we measured the ratio between the number of ECs regulated and the number of ECs featuring the compound as a substrate or product for each compound. Table 4 shows all the compounds that are involved in or regulate >20 EC reactions. Molecules with <4 non-hydrogen atoms are filtered out. The data shown is from *E. coli*, but similar patterns are observed in *S. cerevisiae*, *H. sapiens *and *P. falciparum *[see Additional file 1].

**Table 4:** The regulatory proclivity of common metabolites and regulators in *E. coli*. .

Rank	Compound	Reaction ECs	Regulated ECs	Ratio
1	NAD^+^	116	19	0.16
2	NADPH	82	20	0.24
3	NADH	110	28	0.25
4	NADP^+^	83	22	0.27
5	L-Glutamate	44	12	0.27
6	Acetyl-CoA	33	11	0.33
7	CoA	48	19	0.40
8	ATP	176	70	0.40
9	Pyrophosphate	94	38	0.40
10	Orthophosphate	119	53	0.45
11	ADP	119	70	0.59
12	Pyruvate	42	25	0.60
13	2-Oxoglutarate	22	18	0.82
14	AMP	53	55	1.04
15	UTP	13	22	1.69
16	GTP	20	35	1.75
17	CTP	11	24	2.18
18	L-Cysteine	10	26	2.60
19	Glutathione	9	29	3.22
20	Spermidine	4	22	5.50
21	D-Fructose 1,6-bisphosphate	3	20	6.67
22	Adenosine	3	21	7.00
23	Urea	1	20	20.00
24	Mercaptoethanol	1	40	40.00

Compounds that are either regulated or are involved in more than 20 reactions in *E. coli *are shown. Small molecules (<4 non-hydrogen atoms) are excluded)

The compounds with low regulatory proclivity include NAD(P)^+^/H which has already been mentioned. Glutamate is another molecule which while apparently important in metabolism is not so common in the regulatory network. ATP has a higher ratio than NAD(P)^+^/H. However, when compared to other compounds, ATP does not have a ratio that is remarkably high. The high proclivity compounds are dominated by non-specific regulators such as mercaptoethanol, urea, glutathione and spermidine. D-fructose 1,6-bisphosphate is a common regulator presumably due to its position in a key, but early part of glycolysis. This means it is involved in few reactions but is important for regulating many. Adenosine is also a common regulator, though possibly this is a side-effect of many enzymes being regulated by ATP/ADP which allows them to be regulated by adenosine as well.

### Chemical vs Regulatory Similarity

It is clear from the above analysis that groups of related compounds often regulate the same enzyme. Enzymes regulated by ATP, for instance, are often also regulated by ADP and AMP. The apparently anomalously high regulatory proclivity for adenosine could be a by-product of this. The obvious explanation for this effect is that the determinant of regulator binding is some chemical group common to all these compounds (perhaps the adenosine ring in this case). If this is generally the case then we would expect to see a relationship between the chemical structure similarity of two compounds, and their 'regulatory similarity'. There are many approaches to measuring chemical similarity and in this study we use SIMCOMP[25]. SIMCOMP provides a global similarity score between two compounds based on the size of the common substructures between the two compounds. To measure the regulatory similarity between two compounds we use a binary string comparison measure. Each compound is represented by a string of 1 and 0s, where each position in the string represents an enzyme. If the compound regulates a given enzyme then the enzyme's position in the string is set to 1, otherwise it is set to 0. The strings representing the set of enzymes that two compounds regulate can then be compared using the Jaccard coefficient, as described in the methods section.

**Figure 6:**
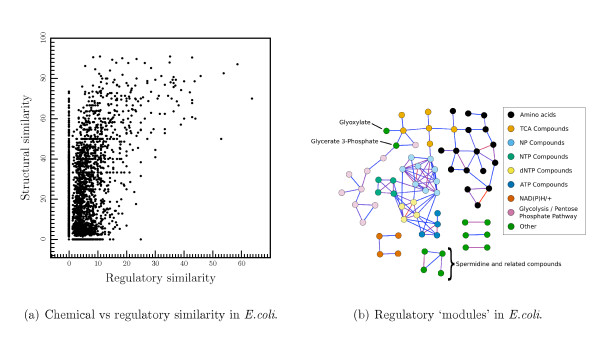
The relationship between the regulatory similarity of a pair of compounds and their chemical similarity. (a) A scatter plot of chemical similarity scores for every pair of compounds in the *E. coli *dataset against regulatory similarity. (b) Graph plot of the *E. coli *regulatory compounds. Each node represents a compound and edges are drawn between nodes when they have a regulatory similarity above 20. Compounds are colored according to the chemical groups or pathways they belong to. Edges are colored such that compounds with the most similar regulatory profiles are connected with red lines and those with less similar ones are connected with blue lines.

Figure 6(a) shows the chemical similarity scores of every possible pair of *E. coli *metabolites against their regulatory similarity. A weak correlation can be seen. Below a chemical similarity score of about 40, there is little regulatory similarity between compounds. However, above a score of 40 several pairs of compounds do show similar regulatory profiles. The correlation between the two values is weak: the Pearson's correlation coefficient is 0.51 (Spearman's rho is 0.16; P-value is 3.3 × 10^-33^).

The most regulatory similar compounds in this dataset are L-valine and L-isoleucine which have a regulatory similarity score of 64. L-valine and L-isoleucine are structurally, functionally and pathway related. Both are derived from 2-(alpha-Hydroxyethyl)thiamine diphosphate in the 'valine, leucine and isoleucine biosynthesis' KEGG pathway (map00290). The other major compound of this pathway, L-leucine, also has high regulatory similarity scores with the other two compounds. Another pair of amino acids that have highly similar regulatory profiles are L-serine and L-glycine. Again these compounds are part of the same pathway ('glycine, serine and threonine metabolism' (map00260)), and indeed are interconverted in a single step catalysed by glycine hydroxymethyltransferase (EC 2.1.2.1).

Other than amino acids, other compounds with similar regulatory profiles are the various nucleotide phosphate compounds. Cytosine phosphate compounds and their uridine counterparts are particularly close as are the various adenosine derived compounds such as ATP and ADP. In contrast, GTP, while chemically similar to ITP has a relatively weak regulatory similarity. The deoxy form of GTP (dGTP) is another example of a compound very close in structure to GTP, but relatively dissimilar in terms of regulation.

It is clear from the pairwise analysis, that there are groups of compounds that regulate similar enzymes due to their similar chemical structures and functional roles. If the modular, pathway-centric view of metabolism is correct we would expect to see compounds within a pathway (or group of pathways) concentrate on regulating enzymes within those pathways and so forming a distinct regulatory unit (as we observe with leucine, valine and isoleucine).

Figure 6(b) shows a graph representation of those compounds in *E. coli *with similar regulatory profiles. Each node represents a compound and edges are drawn between two compounds if their regulatory similarity is above 20. Nodes are then colored to divide them into functionally and structurally similar compounds. The divisions are somewhat arbitrary but they split the compounds into the following chemical and functional groups: amino acids, TCA compounds (any one of the compounds in the main citric acid cycle), glycolysis compounds (any compound involved in glycolysis), NTPs (all nucleoside triphosphate compounds *except *for ATP), dNTPs (all deoxy-nucleoside triphosphate compounds), the ATP group (ATP, ADP, AMP and PO_3_), other NPs (all other nucleoside di and monophosphate compounds), the NAD group (NADH, NAD^+^, NADPH, NADP^+^) and other compounds.

The graph representation reveals a strong grouping of compounds into functional and structural modules that, while hinted at in the pairwise analysis, were not immediately apparent. It is interesting to note that in some cases functional ties are stronger than structural ones. For instance, the various deoxynucleotide triphosphate compounds (dATP, dCTP, dGTP and dTTP) form a tighter group with each other than they do with their respective nucleotide triphosphate compounds (ATP, CTP, GTP and TTP), even though they are chemically closer to them. It is also interesting that the arrangement of the groups of regulators relative to each other mirrors their arrangement in metabolism. Glycolysis compounds, for instance, connect with citric acid cycle compounds in regulation, in the same way as the two metabolic pathways connect. Again, this reflects the importance of chemical structure in defining regulation (and hence these groups), since at the meeting of two metabolic pathways the compounds involved are chemically closely related, it follows that they regulate similar enzymes.

## Discussion

### Remarks on the dataset used

Our knowledge of the metabolic regulatory network is still incomplete. Unlike other networks such as genetic regulation or protein interaction networks there is, to our knowledge, no current technology for performing high-throughput, genome scale measurements of enzyme inhibition/activation. The results we present and the conclusions we draw have to be considered in this light.

An important consequence of the incompleteness in the data is that there is likely to be a bias in the results. Experimental enzymologists are more likely to test a given enzyme for regulation by common molecules such ATP rather than any randomly chosen compound. This will lead to a tendency to over-emphasise the importance of the hub molecules we find in our network. The EcoCyc dataset provides a useful comparison dataset and shows similar patterns, though we cannot rule out the same bias appearing in EcoCyc as well. There does not appear to be any technical way to correct for this bias other than acknowledge it as a caveat in any conclusions we draw.

Another issue with the underlying dataset is that BRENDA (from which the dataset is derived) does not contain easily machine readable information on the type of regulation that a compound performs. This leads to us considering a range of different physical phenomena as one single process, particularly when we consider inhibitors. Enzyme inhibition can be performed by product inhibition, whereby the product simply rebinds to the active site preventing further reactions by competitive inhibition, allosteric or non-competitive inhibition, whereby a molecule binds to a separate regulatory site, and non-specific inhibition whereby a molecule has chemical properties which allow it to disable an enzyme without binding specifically to any site. Ideally we would separate these processes, but this is currently difficult to do in an automatic way. It should be noted that the Eco-Cyc dataset does contain information on regulation type. However the quantity of data available through EcoCyc was found to be smaller.

Finally, it should be recognised that metabolism is a dynamic system. Even in *E. coli*, different enzymes are expressed at different times and so the static networks we analyse here are only an approximation of the true regulatory system. This problem is increased when we consider the network of *H. sapiens*, which, as a multi-cellular organism, has networks which vary not only in time, but from tissue to tissue and from cell to cell.

Clearly a full analysis and modelling of the metabolic regulatory system will require us to know not only the type of regulation that is performed, but also the parameters for each regulation (such as *k*_*i*_). Although BRENDA does include parameters for some interactions, again it is not easily machine readable and so we do not use this information in this study.

### The global architecture of the metabolic and regulatory networks

The analysis of the degree and clustering coefficient distributions shows that the regulatory network has many of the same characteristics as the underlying metabolic network: a power law distribution of node degree leading to a few highly connected hub regulators and a higher clustering coefficient amongst spoke compounds. Networks with these features are thought to be robust and resistant to random node failure since random failures are unlikely to effect the few hub nodes.

In our network, a node failure can be interpreted as a fluctuation in the concentration of a compound above normal operating levels. This could occur by a random mutation leading to the loss of an enzyme required for synthesis of a given compound, a random mutation resulting in constitutive activation of an enzyme (which could lead to an excess of a given compound), the loss of a compound from the environment, or a random environmental event (or hostile organism) could even introduce an excess of a given compound. Organisms with this type of regulatory network architecture are protected against random events (such as mutation) because the loss of any single compound is unlikely to shut down much of the network. Targeted attacks on a hub compound are likely to be highly damaging however.

The lower degree exponent observed in the regulatory network implies that the network is even more skewed towards hub molecules than the underlying metabolic network. However, as we have seen there may well be an inherent bias in the dataset towards these hub molecules. In this light, we cannot ascribe significance to the difference in degree exponent between metabolic and regulatory networks.

The significance of the difference in degree distribution when comparing inhibitors and activators may derive from the usually more specific nature of activatory interactions. If we assume ligand binding causes structural changes in an enzyme which either increase or decrease activity, and given the sensitivity of enzyme active sites to their precise 3D arrangement[26,27], it would seem that there would be many more changes in an active site that would reduce efficiency than there would be that increase it. Inhibition would therefore seem to be an 'easier' task for a compound to perform and to evolve. There are likely to be fewer non-specific activatory compounds with high degree (regulating many enzymes) therefore. It may well also be the case that the non-specific interactions (both inhibitory and activatory) reported in BRENDA are less important *in vivo *than the specific interactions. If that is so, then the topology of the activatory network may better represent the true topology of the whole network.

We have seen how the clustering coefficient in the metabolic network is related to the degree exponent. This leads to a hierarchical architecture whereby the spoke nodes tend to form highly clustered modules that are built up into larger, but more loosely connected modules. Ravasz *et al *observe a very similar pattern including the lowering of the clustering coefficient below a certain degree cutoff[24]. They do not appear to comment on this feature, but presumably it can be interpreted as a fundamental node degree below which there is no apparent hierarchy. For the metabolic network this occurs at a degree of around 10.

We see a similar relationship between *C*(*k*) and *k *in the regulatory network, but with two differences: Firstly the average clustering coefficient is considerably higher (around twice as high). An explanation for the high clustering is that a given compound may have only a small part of its chemical structure recognised at a regulatory site on an enzyme. This means that other chemicals which share the same structural motif can also regulate at the same site and so a cluster of regulators is formed. This may be a useful design feature allowing related molecules within a pathway to feedback and inhibit a common synthesising enzyme (the point of entry into the pathway for instance). This is observed in the synthesis of valine, leucine and isoleucine which share a common structural core that is recognised by the first enzyme in their synthesis pathway: acetolactate synthase. We also observe the process whereby one molecule, such as ATP, inhibits, while a second, related molecule, such as ADP, activates a single enzyme.

The hierarchical clustering appears to breakdown in the regulatory network at a higher degree value than the metabolic network (around 60 compared to 10). The bias commented on above (that hub connections are over represented) means that some connections amongst low *k *molecules may be missing. This will lower the clustering coefficient for low degree compounds and so we cannot rule out this explanation for the discrepancy. Another part of the reason for the higher degree value at which the *C*(*k*) ~ *k*^-1 ^relationship breaks down is the way in which the graph is constructed. To construct the metabolic graph, substrates are connected to products whereas in the regulatory graph, regulators are connected to substrates *and *products. However, even if we divide 60 by two it is still larger than 10 and so this may suggest that the fundamental size of modules controlled by the regulatory network is different to the size of modules in the underlying metabolic network. This would suggest that groups of metabolic pathways or modules tend to be regulated by common compounds.

### Variations in regulatory systems between organisms

Comparison of the degree exponent of the log-log plot for different organism shows that while the two unicellular organisms are relatively similar, the *H. sapiens *network has a slightly higher degree exponent. The effect of this is to make the hub molecules slightly less important in *H. sapiens *than they are in the unicellular organisms. There is no reason to think that there would be changes in the bias between organisms, so we suggest that these results reflect a real difference due to the differing lifestyles of these organisms.

As shown in Table 3, a comparison of those compounds that change in importance between *E. coli *and *H. sapiens *shows some of the local reasons for why this global change takes place. *E. coli *metabolism is regulated more by essential metabolites such as pyruvate, which often act as hub molecules in the regulatory and underlying metabolic networks. In contrast, *H. sapiens *is regulated by molecules whose role in metabolism is relatively small, because they're primarily used as signalling molecules. Transferring information is the role of these molecules rather than metabolic mass or energy.

This reflects the difference between the two organisms lifestyles. An *E. coli *cell must be acutely aware of its environment's energy capacity and ready to quickly respond to changes in that environment (which are often outside its control). The same is true of *S. cerevisiae *cells which showed a similar pattern to *E. coli*. *H. sapiens *cells on the other hand, exist in a strictly controlled medium in which changes are suppressed by regulatory systems acting on a whole body level (the release of glucose by the liver for instance). The *H. sapiens *cells must be ready to alter their metabolism, not in response to changes in energy levels, but to external signals which may tell the cell that the body is preparing for exercise for instance.

We also note that the malarial parasite *P. falciparum *has, in terms of its overall topology, as measured by the power law degree exponent, a network more similar to its host than the other two single celled organisms. Although the data is limited, we would suggest that the parasite has evolved its metabolic regulatory system to match that of *H. sapiens *in order to better survive within the cells and blood stream of its host.

### Variations in regulatory ability between compounds

The observation that some compounds are powerful regulators of metabolism because of their importance *in *metabolism leads to the obvious question: Is the regulatory network simply a copy of the underlying metabolic network?

If so we would expect a linear relationship to be shown in Figure 5. Instead, we find a relatively weak correlation between the importance of a compound in metabolism and its importance in regulation. We suggest that this is often due to the importance of chemical structure. Several small molecules for instance, often seen in metabolism, seem to make poor regulators, perhaps because they diffuse quickly (and hence make good signalers of a cells environment), they make an enzyme using them as regulators over-sensitive to small fluctuations in its environment.

There is also a striking difference between the number of reactions regulated by NAD^+ ^and by ATP, two compounds often referred to as the 'energy currency' of the cell. Although these two compounds are involved in similar numbers of reactions, NAD^+ ^regulates far fewer. To explain this we must look at the common reaction products of these compounds. In metabolism, NAD^+ ^is usually converted into NADH, a change of one proton, while ATP is usually converted into ADP or AMP, a change of one or two phosphate groups. We hypothesise that, due to the small chemical difference between NAD^+ ^and NADH, it is easier for an enzyme to evolve to distinguish ATP from ADP than NAD^+ ^from NADH. To be regulated correctly, an enzyme will need to distinguish the two forms, so we suggest that the chemical structure of NADH/NAD^+ ^is the reason why it is relatively rarely observed as a regulatory molecule.

We also see a slightly weaker relationship between regulatory importance and metabolic importance in *H. sapiens*. This fits in with the conclusion we come to above, that core metabolites are less important in *H. sapiens *metabolic regulation than in *E. coli*. We conclude that while metabolic importance is an important factor in determining whether a compound is an important regulator, the regulatory network is not simply a copy of the metabolic network.

### Compound structure/regulatory similarity

We have seen the importance of chemical structure in understanding metabolic regulation. This links us back to the idea of clustering and modules in the regulatory network. For instance, ATP and ADP regulate many of the same enzymes, because this is both structurally easy to evolve (once an enzyme evolves to bind ATP it is easy to evolve to bind ADP as well) and functionally useful. ATP and ADP are functionally closely related compounds and so being able to respond to changes in the concentration of either compound is useful. Amino acids are another example of a group of compounds that share a structural core and are functionally related, and so we might expect it to be both easy and useful to evolve enzymes that are regulated by sets of them.

Because enzymes need to bind regulator compounds with high specificity, we believe that the whole (or majority) of the regulator's chemical structure is of importance in binding. We have therefore used a global chemical structure similarity method (SIMCOMP) rather than a local similarity method such as the Tanimoto coefficient. The effect of using a different chemical similarity measure will probably be small.

The initial investigation of structural similarity relationship with regulatory similarity in fact shows that the effect is quite weak, as shown in Figure 6(a). Many very similar compounds have quite dissimilar regulatory portfolios. This may be partly explained by incompleteness in the data set. If some regulatory interactions are missing then compounds that should show as similar given complete data, in fact come out as being quite dissimilar.

However, the graph view of the connection data shown in Figure 6(b) shows that functional modules do exist within the regulatory network, and that these often correspond well with chemical structure. Amino acids, for example, form a largely separate group within the graph as do citric acid cycle compounds, glycolysis compounds and nucleotide phosphatides.

Interestingly the connections between these groups in the regulatory network again mirror the underlying metabolic network. Certain amino acids and glycolysis compounds regulate very similar enzymes to citric acid cycle compounds in the same way in which compounds from glycolysis feed into the citric acid cycle and leave to feed into amino acid biosynthesis. This 'flow' of regulation reflects the importance of product negative feedback. Since the product of enzyme reactions feedback to inhibit the enzymes that produced them, structurally similar compounds (that are close in the metabolic network) can also feedback to perform the same regulation.

We can use the relationship between chemical structure similarity and regulatory similarity to predict new regulatory interactions. In the future this would be especially useful for drug compounds where interactions could lead to unwanted side-effects. An extremely simplistic analysis on the KEGG DRUG database[28] shows one way in which this data could be used. We took each compound from DRUG and found all *H. sapiens *metabolites with SIMCOMPP similarity scores above 0.5 to the drug being examined. We then assume that any enzyme regulated in *H. sapiens *by more than 50% of these compounds will also be regulated by the drug compound.

Using this naive method we find 451 potential drug/enzyme interactions. While most are probably not biologically meaningful, we do find some cases which demonstrate the feasibility of the concept. For instance, many testosterone or estrogen derived drugs are linked to EC: 2.3.1.26 (cholesterol acyltransferase). The link between these type of drugs and cholesterol levels is well known, and a few compounds such as hydrocortisone and pregnenolone, are explicitly mentioned in conjunction with cholesterol acyltransferase though not necessarily in the context of enzyme inhibition[29,30]. Another previously known link that we find is cytarabine hydrochloride which is predicted by our analysis, and confirmed in the literature[31], to interact with EC: 3.5.4.5 (cytidine deaminase).

Another more interesting prediction is that valproate, an anti-convulsion drug, inhibits EC: 3.5.3.1 (arginase). The exact molecular mechanism of valproate treatment is currently unknown though it is known to inhibit other enzymes besides arginase[32]. It is also associated with gamma-aminobutyric acid (GABA) and its receptors[33]. GABA is synthesised in 3 steps from ornithine, the product of the arginase catalysed reaction. Furthermore, while the interaction between arginase and valproate has never been directly observed, or to our knowledge predicted, we note that there are two reported cases where patients with arginase disorders were found to be particularly sensitive to valproate medication[34,35].

## Conclusion

The existence of negative feedback loops in the control of metabolic networks is well known[36]. However, a study using this dataset to identify these and other potentially novel control motifs in the regulatory network would help to improve our understanding of the way in which control of the overall network is (or is not) broken up into manageable subunits. Our results suggest that there is a degree of modularity in the regulatory network, but the degree of intra and inter-pathway regulation is an open question that such motif finding methods could help answer. Such studies have been performed on transcription factor and other networks[37].

The linking of this regulatory network with genetic regulation is another area of future work. Clearly both systems often work together to provide correct responses to environmental stimuli. However, there may be cases where one or the other system is preferred, or particular control structures are used which can only be discovered by a combined analysis.

Clearly more data is needed to complete our understanding of the metabolic regulatory network. However, our analysis shows that it shares many of the same properties shown by the metabolic network (and other biological networks), suggesting that common evolutionary mechanisms are responsible for the evolution of the binding of substrates and regulators by enzymes. We also see how both the local and global features of the network differ between *E. coli *and *H. sapiens *reflecting the differences in biology in these two organisms. The role chemical structure plays in determining the suitability of a compound for use as a regulator, and the ability for a single enzyme to be regulated in a common way by whole sets of chemical compounds is also demonstrated.

## Methods

### Data Representation

The metabolic network of an organism can be represented by a bipartite graph in which compounds form one type of node while enzymes form another. The underlying metabolic network is built up by connecting compounds to enzymes by directed edges. Edges from compound nodes to enzyme nodes are used to represent that those compounds are substrates of the enzyme. Edges from enzyme nodes to compounds are used to represent that those compounds are products of the enzyme. Regulatory interactions are represented in our graphs by two further types of labelled edges: representing the inhibition of an enzyme by a compound in the first case, and activation of an enzyme in the second. This graph can be converted to a monopartite network by removing enzyme nodes. In that case, each of the substrate and regulator compounds of an enzyme is connected to each of the enzyme's product compounds (and substrate compounds in the case of regulators).

### Data Sources

The initial metabolic network is built using the KEGG ENZYME and REACTION databases (part of the LIGAND database[28]). ENZYME is used to define those enzymes (EC codes) that are present in a given organism. REACTION is then used to define all the reactions catalysed by each enzyme. All the compounds listed as products or substrates of the extracted reactions are considered metabolites of the given organism, and the metabolic network is built by connecting metabolites to enzymes and enzymes to metabolites according to the substrate/product relationships as described above.

The regulatory information is extracted from the BRENDA database[20]. BRENDA lists compounds known to either inhibit or activate a given EC code and the organism in which the regulation was observed. The textual compound identifiers used in BRENDA are converted to KEGG COMPOUND identifiers in a semi-automatic method. If a KEGG compound is found whose name exactly matches the BRENDA text then that compound is used. If multiple matches are found then a choice is made manually. If no match is found then the compound is discarded. The same method is used for the organisms given in BRENDA which are converted to KEGG organism identifiers. BRENDA entries with the 'More' tag were skipped.

The same procedure was used with the comparison dataset downloaded from Eco-Cyc. We use the 'enzrxns.dat' file from the EcoCyc flatfile distribution. All compounds listed as 'INHIBITORS*' or 'ACTIVATORS*' (where * is blank, -ALLOSTERIC, -COMPETITIVE, -MECHNOTSTATED or -NEITHER) were extracted and converted to KEGG compounds using the same semi-automated method as above.

Note that to remove the many non-natural inhibitors/activators we only examine those regulator compounds that take part in an enzyme reaction as defined by REACTION for the organism in question. Some true, natural metabolites, such as metal ions, may not partake in any enzymatic reactions as substrates/products and the data for these potential regulators is lost.

### Annotation Transfer

For each organism we wish to study, each of the regulatory interactions extracted from BRENDA is considered in turn. Each interaction specifies a KEGG COMPOUND identifier, EC code and the species in which it was observed. If the interaction was observed in the organism we wish to study, then the interaction is used. If the interaction was observed in a different organism from the one under study then all the sequences annotated with the given EC from both organisms are extracted. Each of the sequences from each organism is aligned against each of the sequences from the other organism using BLAST2P[38]. If the organism in which the interaction was observed has multiple sequences annotated with the given EC then the interaction is discarded (there being no way of determining which sequence encodes the form of the enzyme that is regulated). Otherwise the best (lowest E value) hit from the organism under study to the organism in which the interaction was observed is recorded.

We can then filter the results to only include definite regulatory interactions (those observed in the organism under study), high confidence interactions (those observed in organisms with close homologs of the enzyme in the organism under study) and low confidence predictions (those observed in organisms with remoter homologs of the enzyme). An E value cutoff of 10^-40 ^is used in this study. At this cutoff value we can be confident that the annotation transfer occurs between close homologs.

### Data Analysis

All data is stored in a relational database with a Ruby on Rails web front end. Subsequent analyses on the database are largely self-explanatory.

Power law fitting and confidence limit evaluation is performed using the igraph package[39] for the R statistical computing platform. Other analysis is performed using Ruby and the BioRuby[40], RSRuby[41] and Tioga[42] packages. Figure 6(b) was prepared using the Biolayout application[43].

The regulatory similarity between two compounds is defined using the Jaccard similarity of two binary strings. For each compound a binary string is built with each character position in the string representing an enzyme present in the organism under consideration. If the compound is a regulator of an enzyme then the character in the string at that position is 1, otherwise it is 0. The Jaccard similarity, shown in Equation 2 (scaled between 0 and 100 for convenience), between the two strings (the regulatory profiles) then represents how similar the set of enzymes the compounds regulate are. In Equation 2, *M*_11 _is the number of enzymes both compounds are known to regulate, *M*_10 _is the number of enzymes the first compound is known to regulate whilst the second is known not to regulate and *M*_01 _is the number of enzymes the first compound is known not to regulate whilst the second is known to regulate.

J=M11M01+M10+M11×100     (2)
 MathType@MTEF@5@5@+=feaafiart1ev1aaatCvAUfKttLearuWrP9MDH5MBPbIqV92AaeXatLxBI9gBaebbnrfifHhDYfgasaacH8akY=wiFfYdH8Gipec8Eeeu0xXdbba9frFj0=OqFfea0dXdd9vqai=hGuQ8kuc9pgc9s8qqaq=dirpe0xb9q8qiLsFr0=vr0=vr0dc8meaabaqaciaacaGaaeqabaqabeGadaaakeaacqWGkbGscqGH9aqpdaWcaaqaaiabd2eannaaBaaaleaacqaIXaqmcqaIXaqmaeqaaaGcbaGaemyta00aaSbaaSqaaiabicdaWiabigdaXaqabaGccqGHRaWkcqWGnbqtdaWgaaWcbaGaeGymaeJaeGimaadabeaakiabgUcaRiabd2eannaaBaaaleaacqaIXaqmcqaIXaqmaeqaaaaakiabgEna0kabigdaXiabicdaWiabicdaWiaaxMaacaWLjaWaaeWaaeaacqaIYaGmaiaawIcacaGLPaaaaaa@4625@

## Abbreviations

*E. coli*, *Escherichia coli*. *H. sapiens*, *Homo sapiens*. *P. falciparum*, *Plasmodium falciparum*. *S. cerevisiae*, *Saccharomyces cerevisiae*. ATP, adenosine triphosphate. ADP, adenosine diphosphate. AMP, adenosine monophosphate. NAD, nicotinamide adenine dinucleotide. GTP, guanine triphosphate. CTP, cytosine triphosphate. UTP, uridine triphosphate. ITP, inosine triphosphate. TTP, thiamine triphosphate. EC, Enzyme Commission.

## Authors' contributions

AG performed the analysis and conceived of the study. MK and SG helped in the design of the study and in drafting the manuscript. All authors read and approved the final manuscript.

## Supplementary Material

Additional file 1 Supplementary analysis. Description of additional analysis on the dataset, including a comparison of BRENDA with EcoCyc. Click here for file
