# Supplementary material for: Regulation of metabolic networks by small molecule metabolites
Source: BMC Bioinformatics. 2007 Mar 13;8:88. doi: 10.1186/1471-2105-8-88 (PMC1839110; doi:10.1186/1471-2105-8-88)
Supplement: Additional file 1 — Supplementary analysis. Description of additional analysis on the dataset, including a comparison of BRENDA with EcoCyc. [file 1471-2105-8-88-S1.pdf]

Organism	Enzymes	Metabolites
E.coli	703	1229
S.cerevisiae	522	1035
H.sapiens	930	1589
P.falciparum	281	625

Table 1: Numbers of enzymes and metabolites found in the organisms studied. Enzymes are defined as unique EC codes taken from the KEGG GENOME database. Metabolites of an organism are defined as the compounds that are potential substrates or products of all the reactions catalysed by the EC codes found in that organism (taken from the KEGG REACTION database).

Organism	Regulatory Compounds		Regulatory Interactions	
	All	Metabolites	All	Metabolites
E.coli	770	326 (27%)	3602	1847
S.cerevisiae	767	300 (29%)	3049	1462
H.sapiens	923	371 (23%)	3169	1435
P.falciparum	489	175 (28%)	1290	599

Table 2: Numbers of compounds, interactions (compound, EC code pairs) found in BRENDA. Compounds and interactions are divided into metabolite and non-metabolite categories depending on whether the compound is a metabolite of the given organism. The percentage of metabolites found to act as regulators is also given.

1 Supplementary information

A summary of the data sets used is shown in Tables 1 and 2. An E value cutoff of 10^{-40} is used for transferring annotation from one organism to another. The compounds in BRENDA are split into metabolites and non-metabolites based on the enzyme complement of the given organism.

The annotation method used to extract the data from BRENDA uses sequence alignments to transfer annotations across species. The E value cutoff used in these transfers determines the confidence we have in the correctness of the annotation. Table 3 shows the number of different interactions observed in *E.coli* under different E value cutoffs. Native interactions are those observed directly in the species in question.

The relative numbers of metabolite compounds found acting as inhibitors and activators are shown in Table 4 along with the number of inhibitory and activatory interactions. Since a single compound can act as both an inhibitor and activator the compound totals do not necessarily add up to the total number of metabolites given in Table 3. We find that activatory interactions comprise around 20% of the total interactions.

Organism	Regulatory Interactions (Metabolites)				
	Native	10^{-80}	10^{-60}	10^{-40}	10^{-20}
E.coli	1124	1557	1660	1847	2093
S.cerevisiae	452	1046	1209	1462	1640
H.sapiens	527	1329	1379	1435	1510
P.falciparum	5	321	450	599	842

Table 3: The number of metabolite interactions found using various E value cutoffs. 'Native' refers to only those interactions identified in the given organism itself. Otherwise the E value cutoff refers to the maximum E value allowed to transfer annotation between homologous enzymes.

Organism	Regulatory Compounds		Regulatory Interactions	
	Inhibitors	Activators	Inhibitory	Activatory
E.coli	305	130	1497 (81%)	350 (19%)
S.cerevisiae	282	119	1191 (81%)	271 (19%)
H.sapiens	351	115	1123 (78%)	312 (22%)
P.falciparum	166	51	504 (84%)	95 (16%)

Table 4: Number of inhibitor and activator compounds and interactions.

The annotation method leads to a certain degree of overlap between organisms. The Venn diagram in Figure 1(a) shows the amount of overlap between *E.coli*, *S.cerevisiae* and *H.sapiens* interactions at an E value of 10^{-40}. In Figure 1(a) overlapping interactions are defined as those that share the same compound, EC code and common evidence (*i.e.* The interaction was observed in one species and transferred to both of the species we are considering). In Figure 1(b) overlapping interactions are defined as those interactions that share the same compound and EC code. In this case there may be separate evidence for each interaction. *S.cerevisiae* has the most shared interactions with 46% of its interactions also seen in *E.coli* or *H.sapiens* (the majority with *E.coli*). *H.sapiens* as the least overlap with only 23% of interactions shared with the other two model organisms.

An example of the bipartite regulatory network is shown in Figure 2(a) from *E.coli* using 10^{-40}. For clarity, in Figure 2(b) 75% of edges are removed at random. Enzymes are drawn as green circles and compounds as blue filled circles. A clear hub and spoke type network can be seen.

Rank	Enzyme	Interactions
	E.coli	
Rank	Enzyme	Interactions
1	Ribose-phosphate diphosphokinase	36
2	Protein-PII uridyltransferase	31
3	Glutamate synthase (NADPH)	25
4	Glucose-1-phosphate adenyltransferase	23
5	Ornithine carbamoyltransferase	23
6	Pyruvate kinase	21
7	Adenylosuccinate synthase	21
8	Glutaminase	19
9	Asparagine synthase (glutamine-hydrolysing)	19
10	Amidophosphoribosyltransferase	18
	S.cerevisiae	
Rank	Enzyme	Interactions
1	Ribose-phosphate diphosphokinase	35
2	Lactoylglutathione lyase	25
3	1-phosphatidylinositol 4-kinase	22
4	Pyruvate kinase	21
5	Adenylosuccinate synthase	21
6	Ornithine carbamoyltransferase	20
7	Asparagine synthase (glutamine hydrolysing)	19
8	Amidophosphoribosyltransferase	18
9	Hexokinase	18
10	Oxoglutarate dehydrogenase (succinyl-transferring	17
	H.sapiens	
Rank	Enzyme	Interactions
1	Ribose-phosphate diphosphokinase	35
2	Hydroxylacylglutathione hydrolase	30
3	5'-nucleotidase	25
4	Phosphatidate phosphatase	25
5	Phosphodiesterase I	20
6	Pyridoxal kinase	19
7	Inositol oxygenase	19
8	Amidophosphoribosyltransferase	18
9	Oxoglutarate dehydrogenase (succinyl-transferring)	17
10	Diacylglycerol kinase	17
	P.Falciparum	
Rank	Enzyme	Interactions
1	Ribose-phosphate diphosphokinase	33
2	Hydroxylacylglutathione hydrolase	27
3	Lactoylglutathione lyase	22
4	Adenylosuccinate synthase	21
5	1-phosphatidylinositol 4-kinase	21
6	Orotate phosphoribosyltransferase	20
7	Asparagine synthase	19
8	Pyruvate kinase	18
9	Glutamate-ammonia ligase	16
10	Oxoglutarate dehydorgenase	16

Table 5: Ten enzymes regulated by the most number of different compounds in *E.coli*, *S.Cerevisiae*, *H.Sapiens* and *P.Falciparum*.

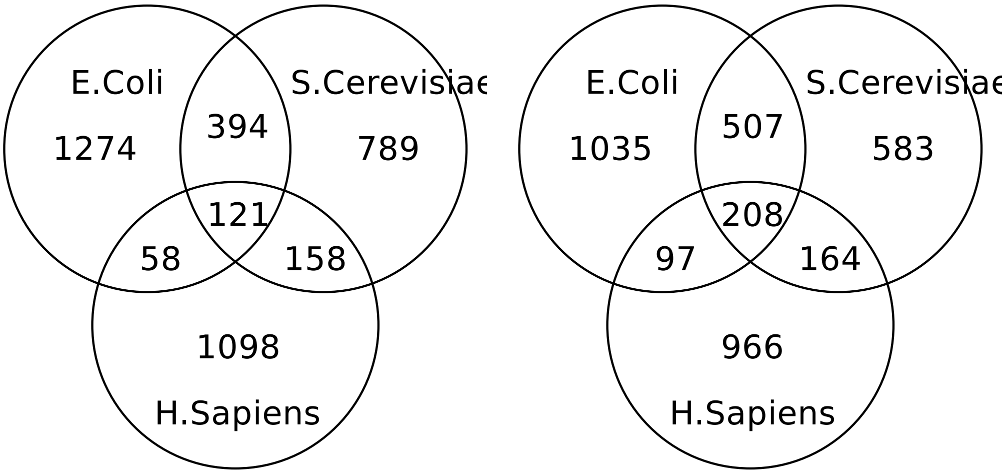

(a) Evidence overlap

(b) Interaction overlap

Figure 1: Venn diagrams of the extent of overlap between the annotations for *E.coli*, *S.cerevisiae* and *H.sapiens*. (a) The overlap where the evidence for a given interaction comes from the same source. (b) The overlap where a given interaction is present in both organisms.

Rank	Compound	Reaction ECs	Regulated ECs	Ratio
1	NAD+	80	11	0.14
2	NADP+	83	12	0.14
3	NADPH	83	15	0.18
4	L-Glutamate	42	8	0.19
5	Acetyl-CoA	30	7	0.23
6	CoA	42	12	0.29
7	NADH	77	25	0.32
8	Pyrophosphate	80	31	0.39
9	ATP	151	65	0.43
10	ADP	103	52	0.50
11	Orthophosphate	101	51	0.50
12	Pyruvate	20	12	0.60
13	2-Oxoglutarate	22	15	0.68
14	AMP	52	50	0.96
15	GDP	19	20	1.05
16	GTP	19	31	1.63
17	CTP	10	22	2.20
18	UTP	11	25	2.27
19	Glutathione	7	24	3.43

Table 6: The regulatory proclivity of common metabolites and regulators in *S.cerevisiae*. Compounds that are either regulated or are involved in more than 20 reactions in *S.Cerevisiae* are shown. Small molecules (<4 non-hydrogen atoms) are excluded).

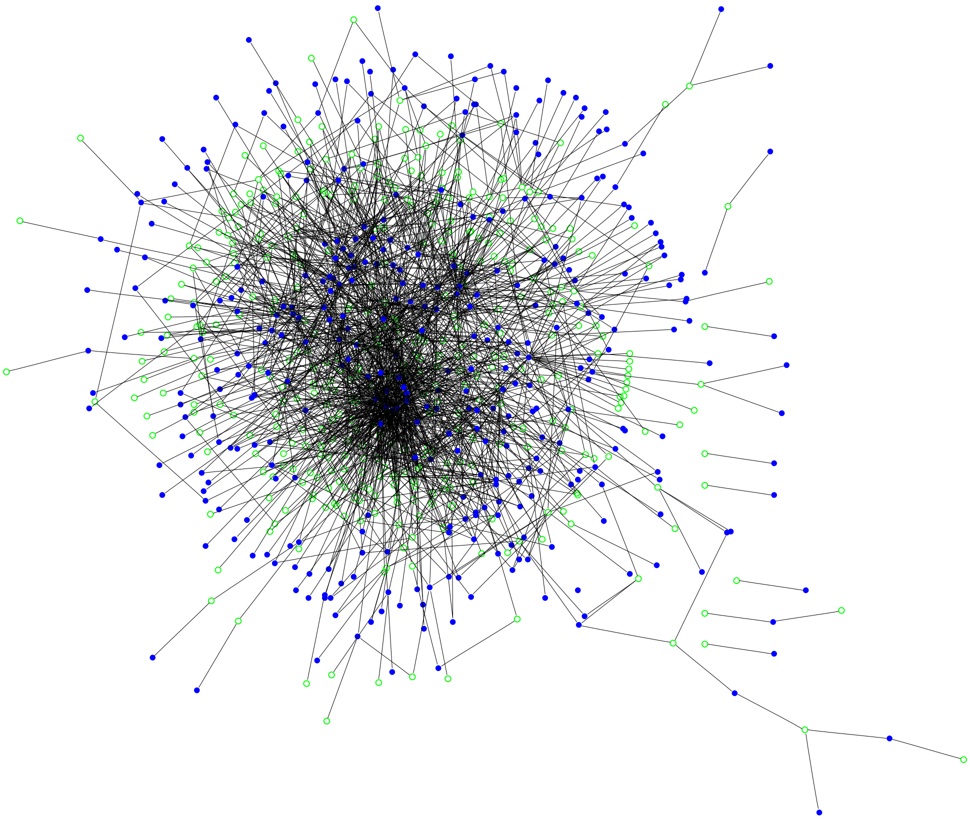

(a) *E.coli* network

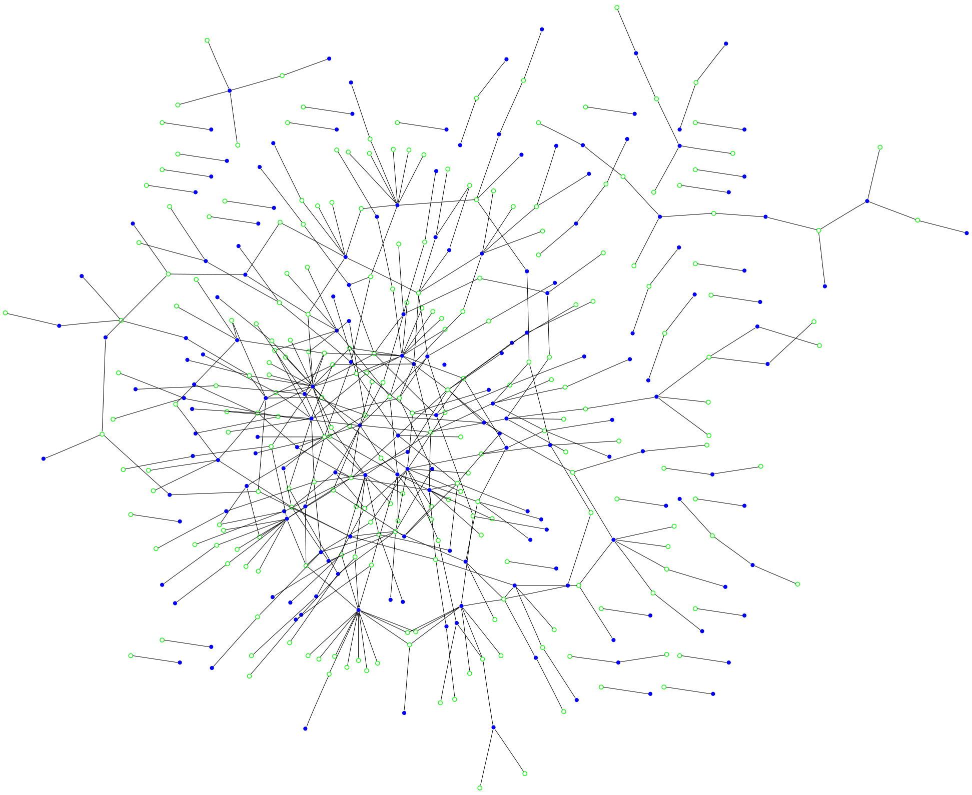

(b) *E.coli* network 25% edges

Figure 2: Bipartite representations of the *E.coli* regulatory network. enzymes are drawn as green circle nodes and compounds as blue filled circle nodes. Edges represent regulatory interactions (inhibition and activation are not distinguished in this figure.

Rank	Compound	Reaction ECs	Regulated ECs	Ratio
1	2-Oxoglutarate	32	3	0.09
2	Acetyl-CoA	33	4	0.12
3	NADPH	115	14	0.12
4	NADP+	118	16	0.14
5	NAD+	116	17	0.15
6	S-Adenosyl-L-homocysteine	27	4	0.15
7	Pyruvate	25	4	0.16
8	CoA	54	9	0.17
9	NADH	106	18	0.17
10	L-Glutamate	46	10	0.22
11	S-Adenosyl-L-methionine	28	7	0.25
12	ATP	175	51	0.29
13	Pyrophosphate	81	24	0.30
14	ADP	125	40	0.32
15	Orthophosphate	118	39	0.33
16	UDP	42	14	0.33
17	AMP	62	31	0.50
18	GDP	26	15	0.58
19	GTP	22	22	1.00
20	UTP	15	23	1.53
21	CTP	10	21	2.10
22	Glutathione	9	30	3.33
23	Mercaptoethanol	1	39	39.00
24	Dithiothreitol	1	51	51.00

Table 7: The regulatory proclivity of common metabolites and regulators in *H.sapiens*. Compounds that are either regulated or are involved in more than 20 reactions in *H.sapiens* are shown. Small molecules (<4 non-hydrogen atoms) are excluded).

Rank	Compound	Reaction ECs	Regulated ECs	Ratio
1	L-Glutamate	23	2	0.09
2	Acetyl-CoA	22	2	0.09
3	NADP+	38	5	0.13
4	CoA	34	5	0.15
5	NAD+	42	7	0.17
6	Pyrophosphate	61	12	0.20
7	Orthophosphate	69	17	0.25
8	NADPH	38	10	0.26
9	NADH	40	11	0.28
10	ATP	111	36	0.32
11	ADP	75	30	0.40
12	AMP	40	23	0.57
13	GTP	18	21	1.17

Table 8: The regulatory proclivity of common metabolites and regulators in *P.falciparum*. Compounds that are either regulated or are involved in more than 20 reactions in *P.falciparum* are shown. Small molecules (<4 non-hydrogen atoms) are excluded).

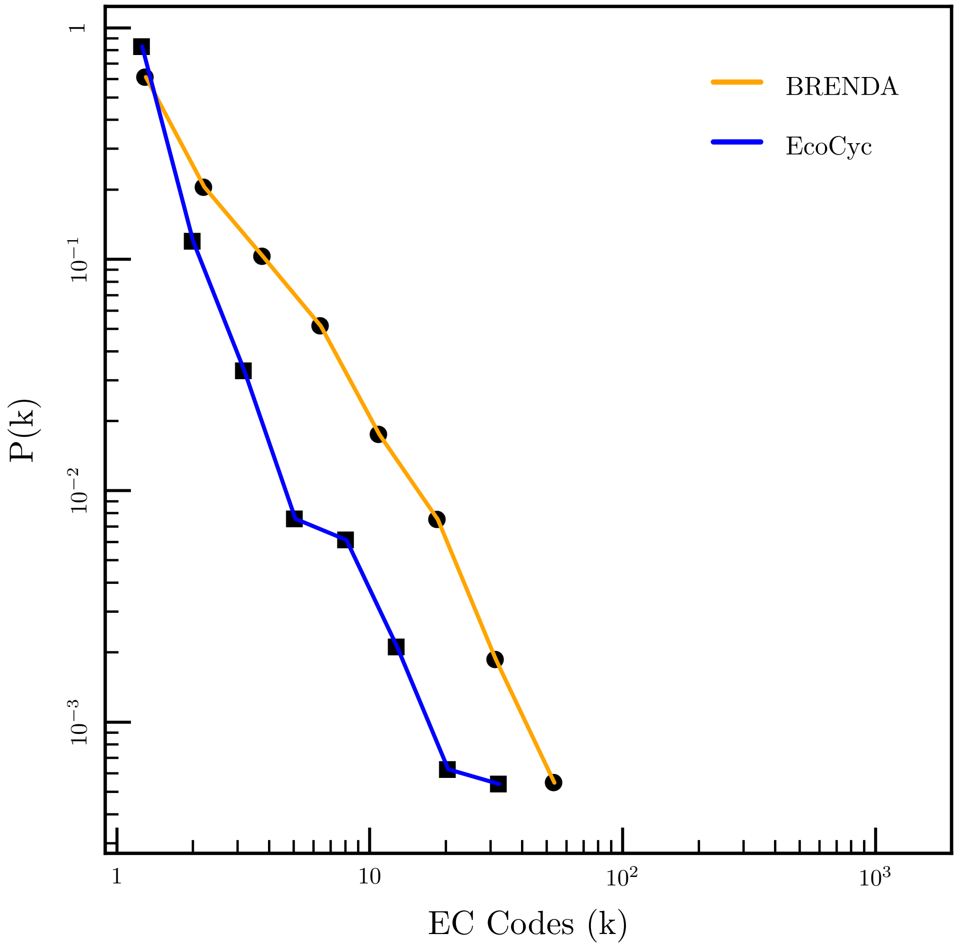

Figure 3: Comparison of the degree distribution between the BRENDA data and equivalent data from EcoCyc. The EcoCyc data shows a similar power law degree distribution. However, the degree exponent is slightly smaller leading to a steeper line. The small size of the EcoCyc data means that we cannot confidently ascribe any significance to the difference.

Rank	Compound
1	ATP
2	ADP
3	AMP
4	PPi
5	GTP
6	NAD
7	NADP
8	Pyruvate
9	HCN
10	NH4

Table 9: The top ten most frequently observed regulatory compounds in *E.coli* found in EcoCyc.

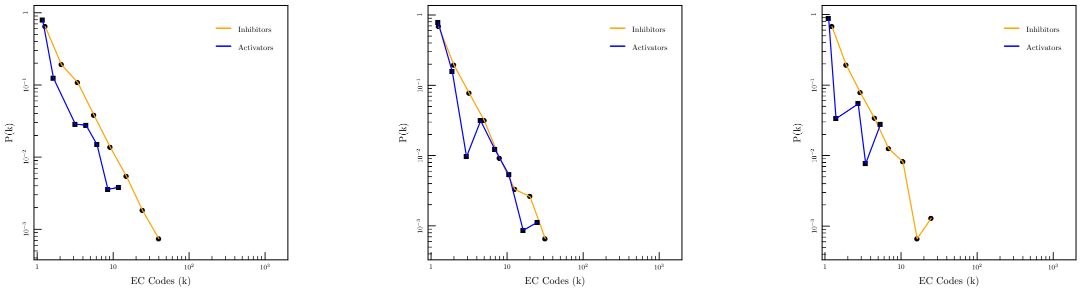

(a) *S.cerevisiae* (b) *H.sapiens* (c) *P.falciparum*

Figure 4: Degree distribution for inhibitors and activators.

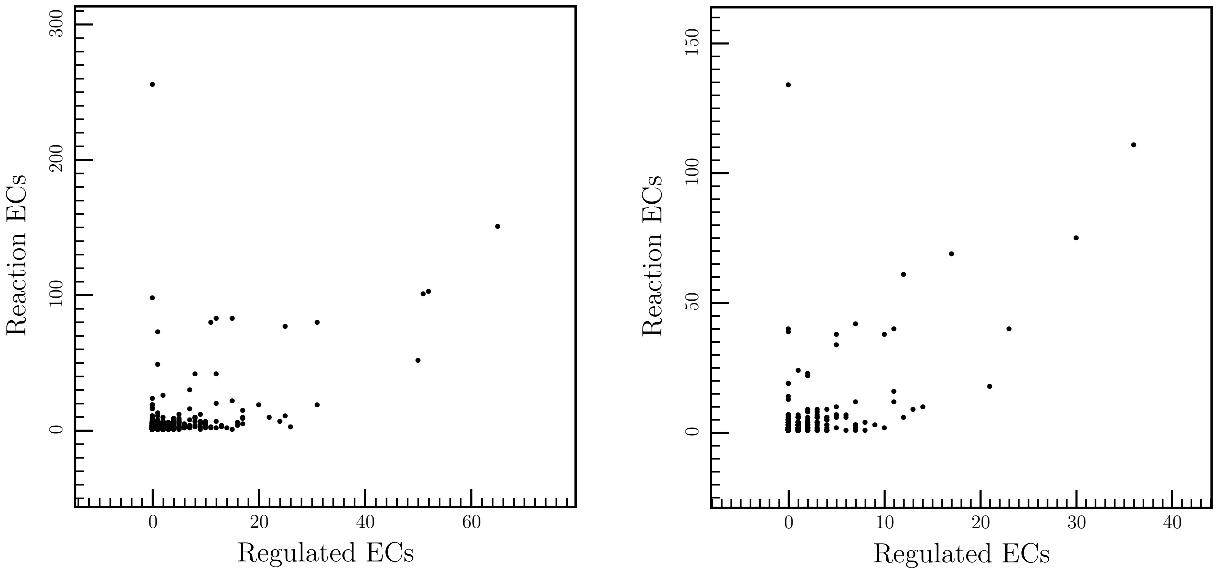

(a) *S.cerevisiae* (b) *P.falciparum*

Figure 5: The relationship between the number of enzymes regulated and metabolised by for metabolite regulators.

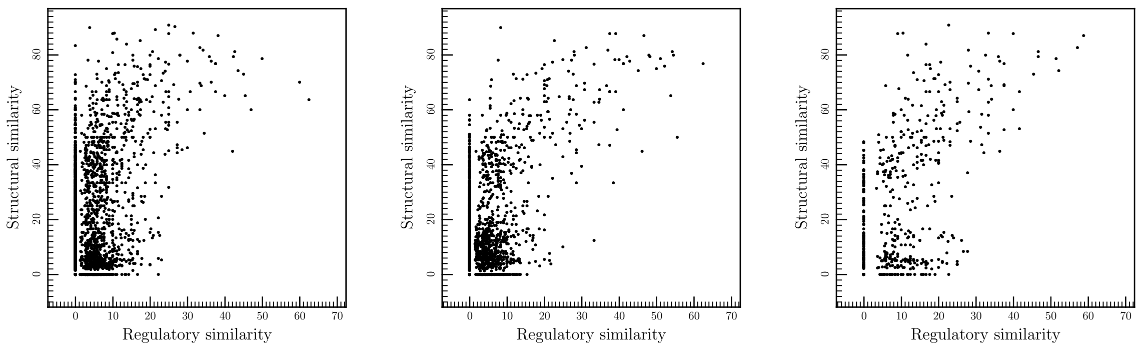

(a) *S.cerevisiae* (b) *H.sapiens* (c) *P.falciparum*

Figure 6: The relationship between chemical structrual similarity and regulatory similarity.
